# Multi-dimensional outcomes following extracorporeal cardiopulmonary resuscitation

**DOI:** 10.1016/j.resplu.2025.100888

**Published:** 2025-01-30

**Authors:** Tharusan Thevathasan, Vanessa Wahl, Joshua Boettel, Megan Kenny, Julia Paul, Sophie Selzer, Abdulla Al Harbi, Eva-Maria Dorsch, Heinrich Audebert, Matthias Rose, Christoph Paul Klapproth, Sonia Lech, Katharina Schmitt, Steffen Desch, Ulf Landmesser, Ralf Westenfeld, Fabian Voss, Carsten Skurk

**Affiliations:** a Department of Cardiology, Angiology and Intensive Care Medicine, Deutsches Herzzentrum der Charité (DHZC), Campus Benjamin Franklin, Berlin, Germany; b Berlin Institute of Health, Berlin, Germany; c Deutsches Zentrum für Herz-Kreislauf-Forschung, Partner Seite Berlin, Berlin, Germany; d Neuroscience Clinical Research Center, Charité -Universitätsmedizin Berlin, Berlin, Germany; e Center for Stroke Research Berlin, Charité-Universitätsmedizin Berlin, Berlin, Germany; f Department of Psychosomatic Medicine, Charité - Universitätsmedizin Berlin, Berlin, Germany; g Department of Psychiatry and Neurosciences, Charité-Universitätsmedizin Berlin, Campus Mitte, Berlin, Germany; h Department of Cardiology, Angiology and Intensive Care Medicine, Deutsches Herzzentrum der Charité (DHZC), Campus Virchow Klinikum, Berlin, Germany; i Department of Internal Medicine/Cardiology, Heart Center Leipzig at the University of Leipzig, Leipzig, Germany; j Division of Cardiology, Pulmonology, and Vascular Medicine, Medical Faculty, University Hospital Düsseldorf, Düsseldorf, Germany

**Keywords:** Cardiac arrest, Extracorporeal cardio-pulmonary resuscitation, Post-resuscitation program, Psychological effect, Myocardial recovery

## Abstract

**Background:**

Recent trials suggested that extracorporeal cardio-pulmonary resuscitation (ECPR) with veno-arterial extracorporeal membrane oxygenation (VA-ECMO) or “ECMELLA” (VA-ECMO plus Impella®) may improve short-term survival and neurological outcomes in selected patients with refractory cardiac arrest. However, long-term effects on cardiac, cognitive, physical and psychological health need further study. A multidisciplinary post-ECPR outpatient care program was developed at two centers, involving cardiologists, neurologists, psychologists and medical sociologists to assess seven key health dimensions.

**Methods:**

This bicentric, multidisciplinary study, conducted from May 2021 to April 2023, included adult ECPR survivors. Outcomes were assessed approximately 22 months post-cardiac arrest, focusing on cardiac, neurological, psychological and multi-organ functions, as well as social, professional and physical performance.

**Results:**

This study included 33 ECPR survivors, who were predominantly male (70%) with a mean age of 55 years. Left-ventricular ejection fraction improved significantly, from 22% during ICU stay to 51% at follow-up in the ECMELLA group and from 31% to 51% in the VA-ECMO group (p = 0.006). Many patients reported dizziness or dyspnea (>52%) during daily activities, with a median New York Heart Association class of 2, EQ-5D-5L score of 53 and elevated NT-proBNP levels. Despite normal neurological scores, 46% had memory issues, 39% struggled with daily organization, 52% had depression and 12% had suicidal thoughts. Physical performance was reduced, with a mean distance of 394 meters in the 6-minute walk test and a 6-minute bicycle ergometry time.

**Conclusion:**

ECPR patients showed significant improvement in left ventricular function over time but their neuropsychological and physical abilities remained compromised. Timely, multidisciplinary rehabilitation is required, starting in the intensive care unit and extending to include psychological support and community reintegration strategies after discharge.

## Introduction

Cardiac arrest remains a major healthcare burden worldwide,^1^, ^2^ which may result in both short- and long-term organ dysfunction in cardiac arrest survivors, including neurologic and cardiac impairments, diminished physical performance, fatigue, depression or suicidal thoughts.^3^ To mitigate reduced blood flow to all organs during refractory cardiac arrest, extracorporeal cardiopulmonary resuscitation (ECPR) by veno-arterial extracorporeal membrane oxygenation (VA-ECMO) has emerged as a treatment strategy with the potential to improve short-term survival and/or neurologic outcome.^4^, ^5^, ^6^ A recent *meta*-analysis across 32 international ECPR centers demonstrated that incorporating left-ventricular (LV) unloading with an Impella® micro-axial flow pump alongside VA-ECMO during ECPR, a concept referred to as “ECMELLA”, may provide short-term survival benefits over VA-ECMO alone.^7^.

Given that ECPR is an exceptionally invasive and costly intervention, there has been criticism from national medical councils regarding the insufficient long-term, patient-centered and coordinated evaluation of its cognitive, physical, social and emotional outcomes.^3^ Critics argued that the long-term effects of ECPR remain unclear. While recent randomized controlled trials (RCTs) have primarily concentrated on short-term survival or neurological outcomes, other observational studies have examined segments of long-term outcomes, such as mortality rate, the Short-Form 36 Survey (SF-36), the Cerebral Performance Category (CPC) scale or the 6-minute walk test (6MWT).^8^, ^9^, ^10^, ^11^, ^12^ These measures may be relatively crude surrogate markers for comprehensively assessing the impact of ECPR on surviving patients' lives. Instead, the International Liaison Committee on Resuscitation (ILCOR) advocated for the evaluation of a standardized framework known as the “Core Outcome Set for Cardiac Arrest” (COSCA). This framework encompasses key domains such as consciousness, cognition, physical symptoms, activities of daily living (ADL), health-related quality of life (QOL), emotional well-being and social participation.^13^.

In accordance with international recommendations by ILCOR and other scientific councils,^3^, ^13^, ^14^ a post-ECPR outpatient care center program was conceptualized at two tertiary care centers employing a holistic, multidisciplinary and multidimensional approach in close collaboration with cardiologists, neurologists, psychologists and medical sociologists. A multidisciplinary, bicentric, mixed-method clinical study was conducted to investigate outcomes across seven dimensions at approximately 22 months after the index cardiac arrest event in adult ECPR survivors. With an exploratory intent, differences in outcomes were compared in patients who had been treated either by VA-ECMO alone or with ECMELLA during the index cardiac arrest event.

## Material and methods

### Study setting and population

This study was performed at two independent and accredited cardiac arrest centers of two tertiary care centers in Berlin and Düsseldorf, Germany.^15^ The pre-registered study (clinicaltrials.gov protocol: NCT05339854) was approved by the Institutional Review Board related to the institutions in which it was performed (protocol: EA4/010/23, May 2023). Data was retrieved retrospectively.

Adult patients were invited for routine clinical follow-up assessment at the newly conceptualized post-ECPR outpatient care center at both tertiary care centers between 20 to 24 months after the ECPR event. ECPR had been provided either with VA-ECMO or with ECMELLA, as previously described.^7^, ^16^ ECPR was conducted in accordance with established eligibility criteria, which included witnessed cardiac arrest, defined “no-flow” and “low-flow” intervals, initial arrest rhythm and the absence of therapeutic limitations or contraindications for VA-ECMO. Left-ventricular unloading with Impella® was performed at the discretion of the treating interventional cardiologists, guided by clinical, hemodynamic or imaging indicators of left-ventricular overload. Patients who died after discharge from the index hospitalization or declined study participation were excluded from the study.

### Conceptualization of the post-ECPR outpatient care center

**Fig. 1:**
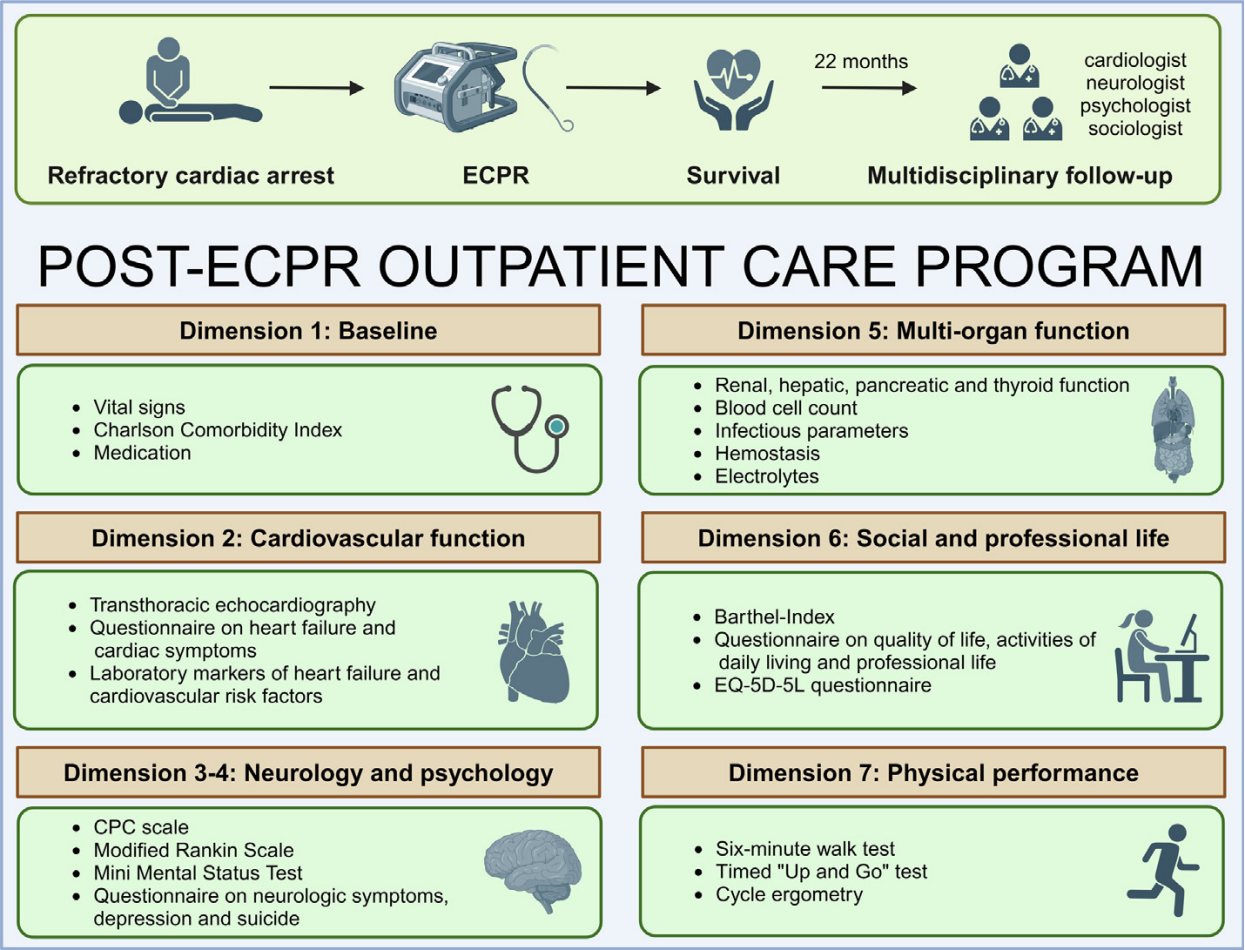
Concept of post-ECPR outpatient care program. The post-ECPR outpatient care program was meticulously developed through a multidisciplinary collaboration with cardiologists, neurologists, psychologists and medical sociologists. The program encompasses seven dimensions of comprehensive, long-term patient assessment, which are derived from the COSCA endorsed by the ILCOR. COSCA, “Core Outcome Set for Cardiac Arrest”; CPC, Cerebral Performance Category; ECPR, extracorporeal cardio-pulmonary resuscitation; ILCOR, International Liaison Committee on Resuscitation. Created in BioRender. https://biorender.com/q80m167

In close collaboration with cardiologists, neurologists, psychologists and medical sociologists, seven dimensions of long-term multidisciplinary patient assessment were conceptualized as part of routine follow-up care after an ECPR event. These dimensions were derived from the COSCA defined by the ILCOR, including “life impact” (such as consciousness, cognition, physical symptoms, ADL, health-related QOL, emotional well-being and participation), “resource use” (such as hospital and ICU length of stay [LOS]) and “pathophysiological manifestations” (such as circulatory function, respiratory function, brain function and adverse events).^13^ At each cardiac arrest center, clinicians assessed the following dimensions during the follow-up examination of each patient (Fig. 1; see Supplemental Text for detailed information):

*Baseline data (dimension I):* Vital signs (ECG, blood pressure, heart rate and oxygen saturation); comorbidities with Charlson Comorbidity Index (CCI); medications.

*Cardiovascular function (dimension II):* Transthoracic echocardiography (TTE) for left-ventricular ejection fraction (LVEF), valvular pathologies, wall motion abnormalities and global longitudinal strain (GLS) via LV strain analysis by three-dimensional speckle tracking; the current LVEF was compared to the LVEF recorded during the index hospitalization after the cardiac arrest event; symptom questionnaires on heart failure and other cardiac symptoms (see Supplemental Material); biomarkers (N-terminal prohormone of brain natriuretic peptide [NT-proBNP], iron levels, creatine kinase [CK] and CK-MB), cardiovascular risk factors (low-density lipoprotein [LDL]-cholesterol, high-density lipoprotein [HDL]-cholesterol, triglycerides, lipoprotein (a) and glycated hemoglobin [HbA1c]); blood gas analysis.

*Neurologic function (dimension III):* CPC scale; modified Rankin Scale (mRS); Mini Mental Status Test (MMST); structured questionnaire on neurologic functions and symptoms (see Supplemental Material). The CPC scale, mRS and MMST are standardized tools to evaluate neurological outcomes, functional disability and cognitive impairment, respectively, in post-resuscitation patients. The structured questionnaire complemented these assessments by capturing detailed patient-reported neurological symptoms and functional deficits for more comprehensive evaluation.

*Psychological function (dimension IV):* a structured questionnaire was utilized to evaluate depression severity and suicide risk (see Supplemental Material). This approach ensured early identification of psychological distress, enabling timely interventions.

*Multi-organ function (dimension V):* Blood tests on renal (creatinine), hepatic (liver enzymes), pancreatic (enzymes) and thyroid (thyroid hormones) functions; blood count; hemostasis; electrolytes; infectious parameters.

*Social and professional life (dimension VI):* Barthel Index; structured questionnaires on ADL, health-related QOL and professional life (see Supplemental Material).

*Physical performance (dimension VII):* 6MWT; timed “Up and Go” test (TUG); cycle ergometry.

*Index cardiac arrest and treatment characteristics.* Information on the index ECPR event and hospitalization were extracted from electronic medical records. Four databases were accessed for hospital data: intensive care electronic patient information system (COPRA System GmbH or CGM Medico) for ICU patient data, Centricity™ RIS-i (GE Healthcare) for interventional data, SAP® ishmed® hospital information system for patient characteristics and hospital data, as well as emergency medical service protocols for cardiac arrest parameters, as detailed in a previous publication.^16^.

### Study outcome

The primary outcome was defined as the assessment of the dimensions II to VII at the time of follow-up examination in the outpatient care center. With an exploratory intent, the primary outcome was compared between patients who had been treated with ECMELLA compared to patients treated with VA-ECMO alone during the index cardiac arrest event.

### Data analysis

Data analysis was performed using R Core Team 2020 (Vienna, Austria). Authors VW and JB had full access to all the data in the study and take responsibility for its integrity and the data analysis. Categorical and continuous variables were compared using Chi^2^ tests and Student’s t-tests (normally distributed) or Fisher’s exact test and Wilcoxon-Mann-Whitney *U* test (non-normally distributed), respectively. Normally distributed continuous variables are expressed as mean (SD), non-normally distributed variables as median (IQR) and categorical variables as frequency (percentage).

## Results

**Fig. 2:**
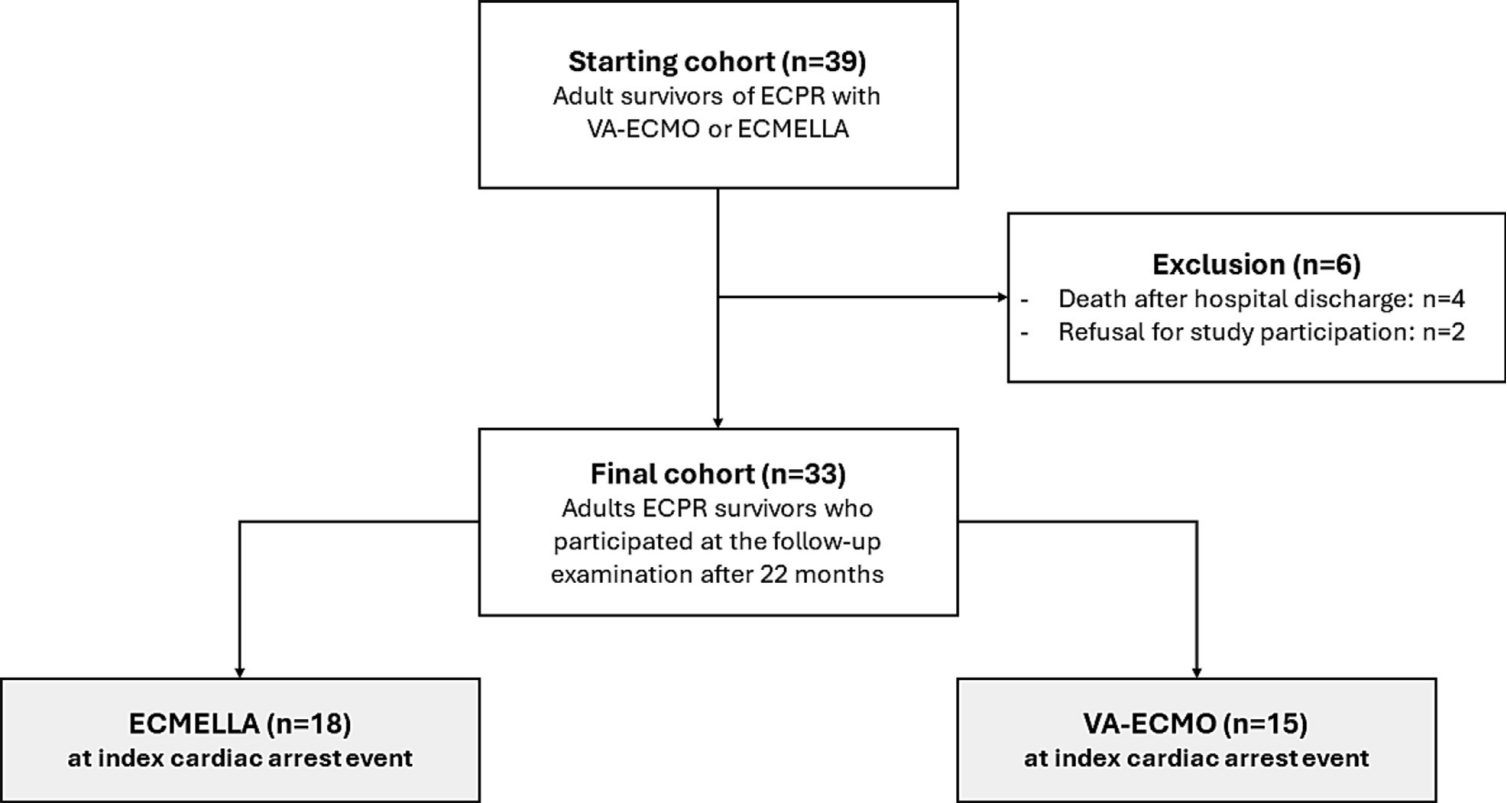
Study subject flow diagram. In total, 39 ECPR survivors were invited to attend the post-ECPR outpatient care center. Six patients were excluded due to death or refusal to participate. Among the 33 participants, 18 had received an ECMELLA device, while 15 had undergone therapy with VA-ECMO only during the initial cardiac arrest event. ECMELLA, combined therapy with VA-ECMO and Impella®; ECPR, extracorporeal cardio-pulmonary resuscitation; VA-ECMO, veno-arterial extracorporeal membrane oxygenation.

In total, 39 ECPR survivors were contacted for reassessment at both post-ECPR outpatient care centers, out of which 33 patients were included in the study. Four patients died after discharge from index hospitalization and 2 patients were excluded due to personal refusal to participate, citing either long distances to the outpatient care center or fear of COVID-19 infection during the pandemic (Fig. 2). The average time of the follow-up assessment was 22 months (±4) after the ECPR event.

The study population predominantly consisted of relatively young patients with a relatively good health status: 23 (70%) male patients with a mean age of 55 (±13) years, mean BMI of 26 (±5) mg/kg^2^ and a low comorbidity level indicated by a CCI of 1 [IQR 1–2] (Table 1). At the index cardiac arrest event, 15 (46%) patients received ECPR by VA-ECMO and 18 (54%) patients by ECMELLA. Acute myocardial infarction (AMI) was the primary cause of refractory cardiac arrest in 22 (67%) patients, with pulmonary embolism (PE) being the second most common cause in 3 (9%) cases. The median “low-flow” time was 43[24–61] minutes and 24 (73%) patients had an initially shockable ECG rhythm. 18 (55%) patients had experienced an out-of-hospital cardiac arrest (OHCA) compared to 15 (45%) patients with an in-hospital cardiac arrest (IHCA). All cardiac arrest cases were witnessed by bystanders, who also performed CPR. The median index hospital and ICU length of stay were 31 [IQR 24–49] and 22 [IQR 15–33] days, respectively. However, these parameters were not statistically different when compared between patients treated with VA-ECMO and those receiving ECMELLA.

**Table 1:** Demographic, cardiac arrest and hospital-level characteristics of patients at the index ECPR event.

Treatment	VA-ECMO (N = 15)	ECMELLA (N = 18)	Total (N = 33)	P-value
Demographic parameters				
Male sex	8 (53.3%)	15 (83.3%)	23 (69.7%)	0.074
Age (years)	53.2 (15.3)	56.6 (11.2)	55.0 (13.2)	0.486
BMI (kg/m^2^)	25.9 (5.54)	26.7 (4.87)	26.3 (5.13)	0.642
Charlson Comorbidity Index	1.00 [0.50–1.50]	1.50 [1.0–2.25]	1 [1.0–2.0]	0.612
Cardiac arrest parameters				
Out-of-hospital cardiac arrest	8 (53.3%)	10 (55.6%)	18 (54.5%)	0.486
Bystander CPR	15 (100%)	18 (100%)	33 (100%)	0.602
Initial shockable ECG rhythm	8 (53.3%)	16 (88.9%)	24 (72.7%)	0.049
Time until ECPR initiation (minutes)	35.0 [25.0–45.0]	57.0 [30.0–62.5]	42.5 [23.8–61.3]	0.208
pH prior ECPR initiation	7.11 (0.261)	7.05 (0.194)	7.08 (0.226)	0.452
Lactate level prior to ECPR initiation (mg/dL)	50.8 (44.4)	63.2 (44.3)	57.6 (44.1)	0.43
Cause of cardiac arrest				
Acute myocardial infarction	6 (40.0%)	16 (88.9%)	22 (66.7%)	0.004
Pulmonary embolism	3 (20.0%)	0 (0.0%)	3 (9.1%)	0.082
Myocarditis	2 (13.3%)	0 (0.0%)	2 (6.1%)	0.164
Drug overdose	1 (6.7%)	0 (0.0%)	1 (3.0%)	0.334
Other	3 (20.0%)	2 (11.1%)	5 (15.2%)	0.693
Hospital-level data				
Hospital length of stay (days)	30.0 [19.5–35.0]	35.0 [28.3–52.8]	31 [24.0–49.0]	0.817
ICU length of stay (days)	18.0 [13.5–30.0]	28.5 [15.3–35.5]	22.0 [15.0–33.0]	0.928
LVEF (during ICU stay) (percent)	30.6 (15.1)	21.6 (11.6)	25.1 (3.5)	0.15

Values are displayed as frequency (percent), mean (standard deviation) or median interquartile range. BMI, body mass index; CPR cardiopulmonary resuscitation; ECG, electrocardiogram; ECMELLA, VA-ECMO plus Impella®; ECPR, extracorporeal cardiopulmonary resuscitation; ICU, intensive care unit; LVEF (left-ventricular ejection fraction); VA-ECMO, veno-arterial extracorporeal membrane oxygenation.

With an exploratory intent, ECMELLA and VA-ECMO only patients were compared, revealing notable differences (Table 1): Patients treated with ECMELLA had more frequently shockable initial ECG rhythms (16 [89%] versus 8 [53%], p = 0.049) and AMI as the causes of cardiac arrest (16 [89%] vs. 6 [40%], p = 0.004). The VA-ECMO group showed more heterogeneous causes of cardiac arrest, including PE (20% of VA-ECMO cases), myocarditis (13%) or drug overdose (7%). Notably, patients with ECMELLA treatment had numerically longer “low-flow” times (57 [IQR 30–63] versus 35 [IQR 25–45] minutes, p = 0.208) and a higher degree of lactic acidosis (pH 7.05 [±0.19] versus 7.11 [±0.26], p = 0.452 and lactate 63 [±44] versus 51 mg/dL [±44.] mg/dL, p = 0.43).

At the follow-up examination, the majority of patients (27 [82%]) exhibited a sinus rhythm (Table 2 and Supplemental Table 1). The resting means blood pressure was relatively low, with systolic values averaging 112 (±24) mmHg and diastolic values averaging 71 (±18) mmHg. The remaining vital signs were unremarkable: average heart rate of 74 beats per minute and peripheral oxygen saturation of 98%.

**Table 2:** Characteristics of patients at follow-up examination in the post-ECPR outpatient care centre.

Treatment		VA-ECMO (N = 15)	ECMELLA (N = 18)	Total (N = 33)	P-value
Transthoracic echocardiography					
LVEF (percent)		51.1 (8.89)	50.7 (9.14)	50.9 (8.87)	0.908
Global longitudinal strain		−15.0% (5.00)	−11.9% (4.68)	−13.2% (4.96)	0.16
Baseline medication					
Any anti-platelet therapy or anticoagulation		12 (80.0%)	15 (83.3%)	27 (81.8%)	0.116
Any heart failure medication		10 (66.7%)	14 (77.8%)	24 (72.7%)	0.116
Heart failure symptoms					
Dyspnoea during daily life		8 (53.3%)	12 (66.7%)	20 (60.6%)	0.454
NYHA class	1	5 (33.3%)	8 (44.4%)	13 (39.4%)	0.811
	2	3 (20.0%)	2 (11.1%)	5 (15.2%)	
	3	6 (40.0%)	6 (33.3%)	12 (36.4%)	
	4	1 (6.7%)	2 (11.1%)	3 (9.1%)	
Angina pectoris at exertion or at rest		5 (33.3%)	6 (33.3%)	11 (33.3%)	1.0
EQ-5D-5L visual analogue scale		52.5 [42.8–67.5]	55.0 [38.8–66.3]	52.5 [40.0–67.5]	0.763
NT-proBNP (ng/L)		1260 (2290)	947 (1680)	1100 (2370)	0.725
Cardiovascular risk factors					
Low-density lipoprotein cholesterol (mg/dL)		102 (65.5)	65.6 (29.4)	83.1 (52.6)	0.065
High-density lipoprotein cholesterol (mg/dL)		62.3 (41.4)	52.1 (22.4)	57.0 (32.8)	0.41
Triglyceride (mg/dL)		125 (57.5)	131 (72.9)	128 (64.5)	0.826
Lipoprotein a (nmol/L)		96.8 (101)	69.1 (85.8)	83.5 (93.3)	0.432
Haemoglobin A1c (percent)		39.1 (7.90)	41.3 (11.6)	40.2 (9.82)	0.541
Cognitive function					
Cerebral Performance Categories scale	1	14 (93.3%)	15 (83.3%)	29 (87.9%)	0.285
	2	1 (6.7%)	0 (0.0%)	1 (3.0%)	
	3	0 (0.0%)	1 (5.6%)	1 (3.0%)	
Modified Rankin scale		1.00 [0.0–2.0]	1.00 [0.75–1.0]	1.00 [0.75–2.0]	0.832
Mini Mental State Examination		29.0 [28.0–30.0]	28.0 [26.5–30.0]	29 [28.0–30.0]	0.111
Psychologic symptoms					
Depressive symptoms	1	5 (33.3%)	5 (27.8%)	10 (30.3%)	0.171
	2	5 (33.3%)	2 (11.1%)	7 (21.2%)	
	3	1 (6.7%)	2 (11.1%)	3 (9.1%)	
	4	0 (0.0%)	5 (27.8%)	5 (15.2%)	
	5	4 (26.7%)	4 (22.2%)	8 (24.2%)	
Thoughts that it would be better to stop being alive	1	1 (6.7%)	3 (16.7%)	4 (12.1%)	0.879
	2	2 (13.3%)	3 (16.7%)	5 (15.2%)	
	3	1 (6.7%)	1 (5.6%)	2 (6.1%)	
	4	2 (13.3%)	3 (16.7%)	5 (15.2%)	
	5	9 (60.0%)	8 (44.4%)	17 (51.5%)	
Precise suicidal thoughts	1	1 (6.7%)	1 (5.6%)	2 (6.1%)	0.926
	2	1 (6.7%)	1 (5.6%)	2 (6.1%)	
	3	0 (0.0%)	1 (5.6%)	1 (3.0%)	
	4	1 (6.7%)	1 (5.6%)	2 (6.1%)	
	5	12 (80.0%)	14 (77.8%)	26 (78.8%)	
Blood examination					
Creatinine (mg/dL)		1.11 (0.494)	1.21 (0.485)	1.16 (0.483)	0.598
Glomerular filtration rate (ml/min)		77.3 (31.2)	73.7 (19.5)	75.5 (25.6)	0.707
Alanine-aminotransferase (U/L)		39.9 (52.7)	33.0 (24.1)	36.4 (40.4)	0.651
Aspartat-aminotransferase (U/L)		32.8 (31.3)	46.4 (72.1)	39.6 (55.1)	0.511
Gamma-glutamyl transferase (U/L)		66.1 (135)	215 (539)	143 (399)	0.299
Bilirubin (mg/dL)		0.477 (0.279)	0.615 (0.341)	0.546 (0.314)	0.234
Alkaline phosphatase (U/L)		148 (219)	129 (127)	138 (176)	0.776
Lipase (U/L)		36.4 (12.6)	70.7 (133)	52.3 (90.9)	0.373
Amylase (U/L)		58.4 (18.7)	69.5 (41.5)	63.3 (30.6)	0.428
Thyroid stimulating hormone (mU/L)		1.51 (0.663)	1.20 (0.561)	1.36 (0.623)	0.18
Haemoglobin (g/dL)		13.9 (1.44)	14.2 (1.45)	14.0 (1.43)	0.55
Leukocytes (x10^9^/L)		8.01 (1.37)	9.71 (3.97)	8.92 (3.12)	0.126
Thrombocytes (x10^3^/µl)		297 (97.4)	222 (44.4)	257 (82.0)	0.016
C-reactive protein (mg/L)		17.8 (55.7)	2.61 (4.33)	9.63 (38.2)	0.329
Partial thromboplastin time (seconds)		34.6 (15.8)	44.5 (54.4)	39.9 (40.9)	0.512
International normalised ratio		1.32 (0.675)	1.12 (0.185)	1.22 (0.496)	0.303
Sodium (mmol/L)		139 (3.60)	140 (2.21)	140 (2.94)	0.673
Potassium (mmol/L)		4.47 (0.353)	4.45 (0.356)	4.46 (0.349)	0.856
Magnesium (mmol/L)		0.888 (0.086)	0.869 (0.899)	0.878 (0.089)	0.553
Calcium (mmol/L)		2.39 (0.061)	2.35 (0.124)	2.37 (0.099)	0.198
Phosphate (mmol/K)		1.12 (0.199)	1.07 (0.273)	1.09 (0.238)	0.585
Activities of daily life					
Limitation in private life due to reduced physical performance	1	5 (33.3%)	5 (27.8%)	10 (30.3%)	0.876
	2	6 (40.0%)	6 (33.3%)	12 (36.4%)	
	3	1 (6.7%)	2 (11.1%)	3 (9.1%)	
	4	0 (0.0%)	1 (5.6%)	1 (3.0%)	
	5	3 (20.0%)	4 (22.2%)	7 (21.2%)	
Limitation in professional life due to reduced physical performances	1	5 (33.3%)	7 (38.9%)	12 (36.4%)	0.83
	2	2 (13.3%)	1 (5.6%)	3 (9.1%)	
	3	0 (0.0%)	1 (5.6%)	1 (3.0%)	
	4	2 (13.3%)	2 (11.1%)	0 (0%)	
	5	6 (40.0%)	7 (38.9%)	4 (12.1%)	
Ability to return to profession	Jobless	5 (33.3%)	7 (38.9%)	12 (36.4%)	0.784
	Employed	3 (20.0%)	3 (16.7%)	6 (18.2%)	
	Retired	7 (46.7%)	7 (38.9%)	14 (42.4%)	
Barthel Index		100 [97.5–100.0]	100 [96.3–100.0]	100 [95.0–100]	0.831
6-minute walk test – total distance (meters)		391 (141)	397 (153)	394 (145)	0.921
Timed „Up and Go“ test – total duration (seconds)		9.36 (3.52)	10.8 (4.02)	10.2 (3.81)	0.305
Cycle ergometry					
Total duration (minutes)		6.1 (2.1)	6.5 (3.1)	6.3 (2.65)	0.651

Values are displayed as frequency (percent), mean (standard deviation) or median interquartile range. Questionnaire scale: 1: fully agree**;** 2: partially agree**;** 3: neutral**;** 4: partially disagree**;** 5: fully disagree.

ECMELLA, VA-ECMO plus Impella®; ECPR, extracorporeal cardiopulmonary resuscitation; LVEF, left-ventricular ejection fraction; NT-proBNP, N-terminal prohormone of brain natriuretic peptide; NYHA, New York Heart Association; VA-ECMO, veno-arterial extracorporeal membrane oxygenation.

### Cardiovascular function

**Fig. 3:**
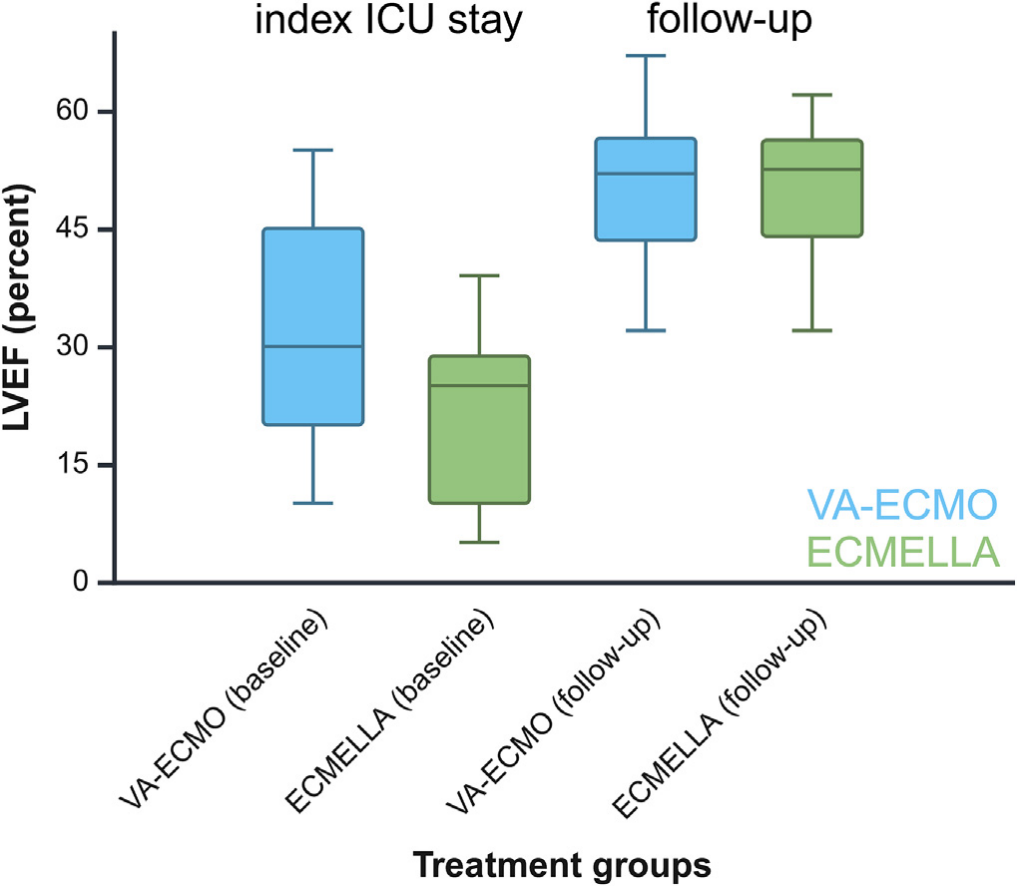
Left-ventricular function after ECPR. Transthoracic echocardiography was conducted by experienced cardiologists to quantify the LVEF both during the initial hospitalization following the cardiac arrest event and at the follow-up examination at the post-ECPR outpatient care center. Patients who received left-ventricular unloading (ECMELLA) exhibited a significantly greater improvement in LVEF compared to those treated with VA-ECMO alone (p = 0.006). Specifically, the LVEF in the ECMELLA group improved from 22% (±12) during the initial ICU stay to 51% (±9) at follow-up, while the LVEF in the VA-ECMO group improved from 31% (±15) to 51% (±9). ECMELLA, combined therapy with VA-ECMO and Impella®; ECPR, extracorporeal cardio-pulmonary resuscitation; ICU, intensive care unit; LVEF, left-ventricular ejection fraction; VA-ECMO, veno-arterial extracorporeal membrane oxygenation.

The LVEF has improved in both treatment groups since the index event, indicating heart failure with improved ejection fraction (HFimPEF): from 25% (±14) during the ICU stay after the index cardiac arrest event to 51% (±9) at the follow-up examination (P = 0.15). However, the mean GLS in the total cohort was abnormal with −13% (±5) at the follow-up examination. Notably, patients treated with LV unloading (ECMELLA) showed a significantly greater improvement in LVEF after the index event compared to VA-ECMO only patients (p = 0.006): The LVEF in the ECMELLA group has improved from 22% (±12) during the index ICU stay to 51% (±9) at the follow-up examination, whereas in the VA-ECMO only group, the LVEF has only improved from 31% (±15) to 51% (±9) (Fig. 3).

Notably, in the total cohort, 20 (61%) patients reported experiencing dyspnea during daily life with a median NYHA class of 2 [IQR 1–3], while 17 (52%) patients reported dizziness. The NT-proBNP level was elevated, averaging 1,100 ng/L (±2,400).

### Multi-organ function

*Neurologic function.* All neurologic tests assessed by objective scores showed unremarkable results: a CPC scale of 1 [IQR 1–1] (normal: 1; severe impairment: 3–4), a mRS of 1 [IQR 0.75–2] (normal: 1; severe impairment ≥ 4) and a MMST score of 29 [IQR 28–30] (normal: ≥28 points; severe impairment: <10 points). However, when asked about their subjective views, 12 (36%) patients felt that their mental capabilities had deteriorated since the ECPR event. Additionally, 15 patients (46%) reported difficulty memorizing new information (Table 2 and Supplemental Table 1).

*Psychological function.* Since the ECPR event, 17 (52%) patients reported experiencing depressive symptoms. Additionally, 9 (27%) patients expressed thoughts that dying would be preferable to living and 4 (12%) had actual suicide plans.

*Multi-organ function.* Renal (creatinine 1.2 mg/dL), hepatic (alanine-aminotransferase 36 U/L and aspartat-aminotransferase 40 U/L), pancreatic (lipase 52 U/L) and thyroid functions (thyroid stimulating hormone 1.4 mU/L), along with blood count (hemoglobin 14 g/dL, thrombocytes 257 x10^3^/µL and 9 leukocytes x10^9^/L), hemostasis (partial thromboplastin time 40 s), electrolytes and infectious parameters (C-reactive protein 10 mg/L), returned to normal or close to normal status since the ECPR event.

### Activities of daily life and physical performance

*Social and professional life.* The objective Barthel Index was excellent with a score of 100 [IQR 95–100] (best value: 100 out of 100 points). However, 22 patients (67%) felt that their private life was limited due to reduced physical performance. Twelve patients (36%) were jobless and 14 patients (42%) were retired. The average EQ-5D-5L score was 53 [IQR 40–68] in the study cohort (best value: 100).

*Physical performance.* Overall physical performance was reduced with a mean distance of 394 m (±145) in the 6MWT (normal distance: 700–800 m) and a mean duration of 6 min (±3) on the cycle ergometer at a maximum power level of 109 Watts (±39). The TUG test results were nearly unremarkable with a mean time of 10 s (±4) (normal: <10 s; severe impairment: ≥30 s). 20 patients (71%) reported that their physical endurance was not the same as before the ECPR event.

## Discussion

ECPR survivors, re-assessed in dedicated multidisciplinary post-ECPR outpatient care centers, demonstrated a recovery of cardiac and objective neurologic functions within 22 months after the index cardiac arrest event. In a subanalysis, the improvement in LVEF was more pronounced in ECMELLA compared to VA-ECMO only patients. However, impairments persisted in subjective neuro-psychological function and physical performance.

ECPR is an increasingly utilized strategy for selected patients with refractory cardiac arrest. It demands substantial resources in terms of clinical staff, time and financial expenses. Despite these extensive expenditures, recent RCTs have shown conflicting results regarding the short-term survival benefits of ECPR.^4^, ^5^, ^6^ To the best of our knowledge, long-term outcomes for ECPR survivors treated with ECMELLA are sparse. Various studies have assessed only fragments of long-term outcomes, such as mortality, health-related QOL or neuro-psychological function.^17^, ^9^, ^10^, ^11^, ^12^ In this study, a systematic, holistic and multidisciplinary approach across seven dimensions was conceptualized by establishing post-ECPR outpatient care centers in close collaboration with different specialties involved in subacute medical care of survivors, such as cardiologists, neurologists, psychologists and medical sociologists. To enable consistent reporting with reduced heterogeneity across medical centers, the COSCA by the ILCOR was used as a standardized outcomes reporting framework for this investigation.

In this study, the patients who survived ECPR and were re-assessed in the post-ECPR outpatient care center, had met the ECPR eligibility criteria during the index event proposed by the Extracorporeal Life Support Organization (ELSO),^18^ including younger age (55 years on average), few comorbidities (average CCI of 1), shockable initial ECG rhythms (approximately 73% of cases) and witnessed cardiac arrest (all included cases) with a presumably reversible cause of cardiac arrest (i.e., approximately 67% with AMI and 9% with PE). Strict selection and treatment in specialized ECPR centers is important to achieve best survival benefits.^12^, ^19^, ^20^ The included ECPR cases were treated at specialized tertiary care centers certified for cardiac arrest treatment, which have shown improved outcomes over recent years.^15^ Information on patients who refused to attend the outpatient care center was unavailable despite multiple telephone and postal follow-up attempts. Nevertheless, the patients who attended the outpatient care center showed good recovery across multiple dimensions.

Notably, the LV function recovered significantly better in the ECMELLA compared to the VA-ECMO group between ICU admission and follow-up assessment in the outpatient care center. This improvement is presumably due to better early coronary perfusion and reduced LV wall strain provided by the Impella®, which might have facilitated less myocardial and systemic ischemia and improved LV recovery and support. It is important to acknowledge that these explanations remain speculative, as mechanistic evidence in humans or data from RCTs are currently lacking. Recent studies, including the first large RCT in cardiogenic shock and the first *meta*-analysis of ECPR patients,^7^, ^21^ have shown survival benefits with Impella® use, highlighting its potential advantages in AMI-induced cardiogenic shock and refractory cardiac arrest. Despite this, the indication and criteria for LV unloading during ECPR remain to be clarified. The observation of almost fully recovered LV function in ECPR patients in this study emphasizes the need for further investigation. This study suggests that cardiac function might be reversible in the long-term if patients can overcome the “vulnerable” period immediately after the ECPR event. Impella® might be a useful temporary ventricular assist device to bridge this “vulnerable” period by unloading the LV thereby counteracting the detrimental effects of VA-ECMO.^22^ It is important to acknowledge that patients receiving EMCELLA therapy predominantly presented with different underlying etiologies of cardiac arrest, primarily AMI, whereas those treated exclusively with VA-ECMO were more commonly diagnosed with conditions such as PE and myocarditis.

Although, patients in both groups showed good recovery in LVEF, they still presented with heart failure symptoms, including exhaustion in daily life and dyspnea (mostly NYHA classes II or III), dizziness, nocturia or leg oedema. Additionally, they exhibited elevated serum markers (such as NT-proBNP) and reduced general health status (53 points on EQ-5D-5L form) in both treatment groups. Physical tests also showed impaired results. Despite the observed good LV recovery, clinical symptoms and physical impairment may not recede completely after surviving ECPR. A recent *meta*-analysis indicated that ICU patients lose approximately two percent of muscle mass per ICU day during the first week,^23^ which might impair recovery and independence for years following the index event.^24^ ECPR patients in this study spent more than three weeks in the ICU and are considered some of the most severely ill patients in the cardiac ICU due to their complex therapy with ECMO or ECMELLA, targeted temperature management, mechanical ventilation, renal replacement therapy and antibiotic therapy if required. Therefore, cardiac rehabilitation after the index ECPR event, including early mobilization, physical therapy, lifestyle education and inclusion in heart failure networks, are important measures to facilitate long-term improvements. Though, inserted ECMO and Impella® cannulas might prohibit early sedation weaning or mobilization.^25^.

Although the neurologic function recovered in the majority of patients according to objective scores (normal CPC scale, mRS scale and MMST), many patients reported subjective issues with concentration, memory, planning and organization in structured questionnaires. Remarkably, psychological issues, including anxiety, depression and suicidal thoughts, appear to be significant concerns among ECPR survivors, corroborating findings from studies on cardiac arrest survivors conducted years ago.^26^ The aforementioned physical impairments might lead to limitations in private (67% of patients in this study) and professional (46% of patients in this study) life, which could further exacerbate psychological stress. However, these issues seem to primarily affect interactions with other people, as ECPR survivors were able to perform self-dependent activities of daily living, such as eating, grooming or toilet use, as indicated by the normal Barthel Index. ICUs are stressful environments where patients, often isolated in communication, may be exposed to pain, nausea, vomiting, fatigue, thirst and delirium due to invasive medical procedures, potent drugs, medically unstable situations and noisy or busy surroundings. Consequently, ICU survivors might experience pain, depression and post-traumatic stress disorder after discharge.^27^, ^28^ Apart from medical and physical therapy, these findings underscore the urgent need for close psychological co-treatment and social reintegration of ECPR patients. Screening for psychological disorders and early intervention might be necessary.

This study has certain limitations. First, presumably, only patients with good general performance were able to attend the post-ECPR outpatient care center. However, all ECPR survivors were invited through telephone calls or postal invitations. Only two patients refused to participate in this study, and there is no information regarding these patients, which may introduce selection bias. Second, patients were in contact with most of the medical personnel for the first time, which may have affected their openness in answering questions on sensitive topics such as suicidal thoughts. Nonetheless, a trustful environment was facilitated for each patient and each patient received a list of all questions prior to the conversation. Third, the study was conducted at two experienced cardiac arrest centers with elaborate resources for multidisciplinary post-ECPR care and rehabilitation. The included study cohort was relatively small, given the high mortality rate after ECPR. These findings might not be applicable to smaller medical centers with less extensive post-ECPR infrastructures.

## Conclusion

This study revealed substantial improvements in LV function of ECPR survivors, particularly among ECMELLA-treated patients, who demonstrated significantly greater cardiac recovery compared to VA-ECMO-only patients. However, long-term follow-up highlighted prevalent psychological challenges, including depressive symptoms and reduced QOL, alongside physical endurance limitations. This study underscores the necessity for close and early multidimensional and multidisciplinary rehabilitation, beginning in the ICU, and incorporating psychological co-treatment and social reintegration after hospital discharge. Focusing only on limited long-term outcomes, such as mortality or CPC scale, does neither capture the complete aftermath of ECPR nor indicates its success.

## Funding sources

This research did not receive any specific grant from funding agencies in the public, commercial or not-for-profit sectors.

## Data availability statement

The data underlying this article cannot be shared publicly due to the privacy of individuals that participated in the study. The data will be shared on reasonable request to the corresponding author.

## CRediT authorship contribution statement

**Tharusan Thevathasan:** Writing – review & editing, Writing – original draft, Visualization, Validation, Supervision, Resources, Project administration, Methodology, Investigation, Formal analysis, Data curation, Conceptualization. **Vanessa Wahl:** Writing – review & editing, Visualization, Methodology, Investigation, Formal analysis, Data curation. **Joshua Boettel:** Writing – review & editing, Methodology, Investigation, Data curation. **Megan Kenny:** Writing – review & editing, Validation, Methodology, Investigation. **Julia Paul:** Writing – review & editing, Validation, Methodology, Investigation, Data curation. **Sophie Selzer:** Writing – review & editing, Resources, Methodology, Investigation, Data curation. **Abdulla Al Harbi:** Writing – review & editing, Validation, Resources, Methodology, Investigation, Data curation. **Eva-Maria Dorsch:** Writing – review & editing, Validation, Resources, Methodology, Investigation, Conceptualization. **Heinrich Audebert:** Writing – review & editing, Validation, Resources, Methodology, Investigation, Data curation, Conceptualization. **Matthias Rose:** Writing – review & editing, Validation, Resources, Methodology, Investigation. **Christoph Paul Klapproth:** Writing – review & editing, Validation, Methodology, Investigation. **Sonia Lech:** Writing – review & editing, Validation, Resources, Methodology, Investigation, Conceptualization. **Katharina Schmitt:** Writing – review & editing, Validation, Resources, Methodology, Investigation. **Steffen Desch:** Writing – review & editing, Validation, Resources, Methodology, Investigation, Data curation. **Ulf Landmesser:** Writing – review & editing, Validation, Resources, Project administration, Methodology, Investigation, Data curation, Conceptualization. **Ralf Westenfeld:** Writing – review & editing, Validation, Resources, Methodology, Investigation, Data curation, Conceptualization. **Fabian Voss:** Writing – review & editing, Validation, Resources, Project administration, Methodology, Investigation, Data curation, Conceptualization. **Carsten Skurk:** Writing – review & editing, Validation, Resources, Methodology, Investigation, Data curation, Conceptualization.

## Declaration of competing interest

The authors declare that they have no known competing financial interests or personal relationships that could have appeared to influence the work reported in this paper.

## References

[1] Gräsner J.T., Herlitz J., Tjelmeland I.B.M. (2021). European Resuscitation Council Guidelines 2021: Epidemiology of cardiac arrest in Europe. Resuscitation.

[2] Holmberg M.J., Ross C.E., Fitzmaurice G.M. (2019). Annual incidence of adult and pediatric in-hospital cardiac arrest in the United States. Circ Cardiovasc Qual Outcomes.

[3] Sawyer K.N., Camp-Rogers T.R., Kotini-Shah P. (2020). Sudden cardiac arrest survivorship: a scientific statement from the American heart association. Circulation.

[4] Yannopoulos D., Bartos J., Raveendran G. (2020). Advanced reperfusion strategies for patients with out-of-hospital cardiac arrest and refractory ventricular fibrillation (ARREST): a phase 2, single centre, open-label, randomised controlled trial. Lancet.

[5] Belohlavek J., Smalcova J., Rob D. (2022). Effect of intra-arrest transport, extracorporeal cardiopulmonary resuscitation, and immediate invasive assessment and treatment on functional neurologic outcome in refractory out-of-hospital cardiac arrest: a randomized clinical trial. JAMA.

[6] Suverein MM, Delnoij TSR, Lorusso R, et al. Early Extracorporeal CPR for Refractory Out-of-Hospital Cardiac Arrest. https://DoiOrg/101056/NEJMoa2204511 2023;388:299–309. doi: 10.1056/NEJMOA2204511. 10.1056/NEJMoa2204511 36720132

[7] Thevathasan T., Füreder L., Fechtner M. (2024). Left-ventricular unloading with impella during refractory cardiac arrest treated with extracorporeal cardiopulmonary resuscitation: a systematic review and meta-analysis. Crit Care Med.

[8] Spangenberg T., Schewel J., Dreher A. (2018). Health related quality of life after extracorporeal cardiopulmonary resuscitation in refractory cardiac arrest. Resuscitation.

[9] Lang C.N., Schroth F., Zotzmann V. (2019). Good long term quality of life after emergency extracorporeal life support for cardiogenic shock and extracorporeal cardiopulmonary resuscitation. Resuscitation.

[10] Alexy T., Kalra R., Kosmopoulos M. (2023). Initial hospital length of stay and long-term survival of patients successfully resuscitated using extracorporeal cardiopulmonary resuscitation for refractory out-of-hospital cardiac arrest. Eur Hear Journal Acute Cardiovasc Care.

[11] Kikuta S., Inoue A., Ishihara S. (2023). Long-term outcomes and prognostic factors of extracorporeal cardiopulmonary resuscitation in patients older than 75 years: a single-centre retrospective study. Emerg Med J.

[12] Gregers E., Linde L., Kunkel J.B. (2024). Health-related quality of life and cognitive function after out-of-hospital cardiac arrest; a comparison of prehospital return-of-spontaneous circulation and refractory arrest managed with extracorporeal cardiopulmonary resuscitation. Resuscitation.

[13] Haywood K., Whitehead L., Nadkarni V.M. (2018). COSCA (Core Outcome Set for Cardiac Arrest) in adults: an advisory statement from the international liaison committee on resuscitation. Circulation.

[14] Perkins G.D., Jacobs I.G., Nadkarni V.M. (2015). Cardiac arrest and cardiopulmonary resuscitation outcome reports: update of the Utstein Resuscitation Registry Templates for Out-of-Hospital Cardiac Arrest: a statement for healthcare professionals from a task force of the International Liaison Committee on Resuscitation (American Heart Association, European Resuscitation Council, Australian and New Zealand Council on Resuscitation, Heart and Stroke Foundation of Canada, InterAmerican Heart Foundation, Resuscitation Council of Southern Africa, R. Circulation.

[15] Voß F., Thevathasan T., Scholz K.H. (2023). Accredited cardiac arrest centers facilitate eCPR and improve neurological outcome. Resuscitation.

[16] Thevathasan T., Kenny M.A., Krause F.J. (2023). Left-ventricular unloading in extracorporeal cardiopulmonary resuscitation due to acute myocardial infarction - A multicenter study. Resuscitation.

[17] Rasalingam Mørk S., Qvist Kristensen L., Christensen S., Tang M., Juhl Terkelsen C., Eiskjær H. (2023). Long-term survival, functional capacity and quality of life after refractory out-of-hospital cardiac arrest treated with mechanical circulatory support. Resusc plus.

[18] Richardson A.C., Tonna J.E., Nanjayya V. (2021). Extracorporeal cardiopulmonary resuscitation in adults. Interim guideline consensus statement from the extracorporeal life support organization. ASAIO J.

[19] Ubben J.F.H., Heuts S., Delnoij T.S.R. (2023). Extracorporeal cardiopulmonary resuscitation for refractory OHCA: lessons from three randomized controlled trials-the trialists’ view. Eur Hear Journal Acute Cardiovasc Care.

[20] Gregers E., Mørk S.R., Linde L. (2022). Extracorporeal cardiopulmonary resuscitation: a national study on the association between favourable neurological status and biomarkers of hypoperfusion, inflammation, and organ injury. Eur Hear Journal Acute Cardiovasc Care.

[21] Møller J.E., Engstrøm T., Jensen L.O. (2024). Microaxial flow pump or standard care in infarct-related cardiogenic shock. N Engl J Med.

[22] Esposito M.L., Zhang Y., Qiao X. (2018). Left ventricular unloading before reperfusion promotes functional recovery after acute myocardial infarction. J Am Coll Cardiol.

[23] Fazzini B., Märkl T., Costas C. (2023). The rate and assessment of muscle wasting during critical illness: a systematic review and meta-analysis. Crit Care.

[24] Herridge M.S., Cheung A.M., Tansey C.M. (2003). One-year outcomes in survivors of the acute respiratory distress syndrome. N Engl J Med.

[25] Hayes K., Holland A.E., Pellegrino V.A., Mathur S., Hodgson C.L. (2018). Acute skeletal muscle wasting and relation to physical function in patients requiring extracorporeal membrane oxygenation (ECMO). J Crit Care.

[26] Wilder Schaaf K.P., Artman L.K., Peberdy M.A. (2013). Anxiety, depression, and PTSD following cardiac arrest: a systematic review of the literature. Resuscitation.

[27] Parker A.M., Sricharoenchai T., Raparla S., Schneck K.W., Bienvenu O.J., Needham D.M. (2015). Posttraumatic stress disorder in critical illness survivors: a metaanalysis. Crit Care Med.

[28] Rabiee A., Nikayin S., Hashem M.D. (2016). Depressive symptoms after critical illness: a systematic review and meta-analysis. Crit Care Med.

