# Supplementary material for: Multi-dimensional outcomes following extracorporeal cardiopulmonary resuscitation
Source: Resusc Plus. 2025 Jan 30;22:100888. doi: 10.1016/j.resplu.2025.100888 (PMC11850733; doi:10.1016/j.resplu.2025.100888)
Supplement: Supplementary Data 1 [file mmc1.docx]

**Supplemental Materials**

**Dimensions of assessment in the outpatient care center**

*Baseline data (dimension I).* The patient's vital parameters, such as 12-lead electrocardiogram (ECG), arterial blood pressure, heart rate and peripheral oxygen saturation were measured at rest. The comorbidity level of each patient was assessed using the *Charlson Comorbidity Index* (CCI).[1] Additionally, the patient's medication list was documented.

*Cardiovascular function (dimension II).* Transthoracic echocardiography (TTE) was performed by experienced cardiologists to quantify the left-ventricular ejection fraction (LVEF), valvular pathologies, wall motion abnormalities and global longitudinal strain (GLS) via LV strain analysis by three-dimensional speckle tracking. The patient's current LVEF was compared to the LVEF recorded during the index hospitalization after the cardiac arrest event, which was retrieved from electronic medical records. Additionally, patients completed structured symptom questionnaires on heart failure and other cardiac symptoms (see Supplemental Material below). Serum biomarkers of heart failure and cardiac enzymes were measured, including N-terminal prohormone of brain natriuretic peptide (NT-proBNP), iron levels, creatine kinase (CK) and CK-MB, as well as venous blood gas samples. Cardiovascular risk factors were also assessed, including low-density lipoprotein (LDL)-cholesterol, high-density lipoprotein (HDL)-cholesterol, triglycerides, lipoprotein (a) and glycated hemoglobin (HbA1c).

*Neurologic function (dimension III).* Cognitive function was evaluated by the CPC scale,[3] the modified Rankin Scale (mRS)[4] and the Mini Mental Status Test (MMST).[5] Additionally, a structured questionnaire was utilized to assess neurologic function and symptoms (see Supplemental Material below).

*Psychological function (dimension IV).* Symptoms of depression and suicide parameters were evaluated using a structured questionnaire (see Supplemental Material below).

*Multi-organ function (dimensions V).* Blood samples were collected to assess renal, liver, pancreatic and thyroid functions. This evaluation also included analysis of blood count, hemostasis, electrolytes and infectious parameters.

*Social and professional life (dimension VI).* The Barthel Index[6] and structured questionnaires on ADL, health-related QOL and the professional life were employed (see Supplemental Material below), including the EuroQol-5 Dimension 5-Level (EQ-5D-5L) questionnaire.[2]

*Physical performance (VII).* The patients´ physical capacities were tested with the 6MWT,[7] timed “Up and Go” test (TUG)[8] and cycle ergometry.[9]

**Questionnaire on neuro-psychological functions**

1. Have you had a new stroke/transient ischemic attack since the cardiac arrest event?

| □ Yes | □ No |
| --- | --- |

1. Now I have the same ability to concentrate as prior to the cardiac arrest event.

| □ Fully agree | □ Partially agree | □ Neutral | □ Partially disagree | □ Fully disagree |
| --- | --- | --- | --- | --- |

1. I have trouble to memorize new information since the cardiac arrest event.

| □ Fully agree | □ Partially agree | □ Neutral | □ Partially disagree | □ Fully disagree |
| --- | --- | --- | --- | --- |

1. I have trouble to remember old information since the cardiac arrest event.

| □ Fully agree | □ Partially agree | □ Neutral | □ Partially disagree | □ Fully disagree |
| --- | --- | --- | --- | --- |

1. I have trouble to make plans and to execute plans since the cardiac arrest event.

| □ Fully agree | □ Partially agree | □ Neutral | □ Partially disagree | □ Fully disagree |
| --- | --- | --- | --- | --- |

1. I need help with planning or organizing my daily life now.

| □ Fully agree | □ Partially agree | □ Neutral | □ Partially disagree | □ Fully disagree |
| --- | --- | --- | --- | --- |

1. My mental capabilities since the cardiac arrest event have…

| □ Strongly improved | □ Moderately improved | □ Did not changed | □ Moderately deteriorated | □ Strongly deteriorated |
| --- | --- | --- | --- | --- |

1. Are you working, jobless or retired now?

| □ I am working | □ I am jobless | □ I am retired |
| --- | --- | --- |

1. If you are working: I work as ______________________________________.

I worked as ____________________________ prior the cardiac arrest event.

1. The performance in my professional life is similar to prior to the cardiac arrest event.

| □ Fully agree | □ Partially agree | □ Neutral | □ Partially disagree | □ Fully disagree |
| --- | --- | --- | --- | --- |

1. I have depressive symptoms since the cardiac arrest event.

| □ Fully agree | □ Partially agree | □ Neutral | □ Partially disagree | □ Fully disagree |
| --- | --- | --- | --- | --- |

1. I have thoughts that it would be better to stop being alive since the cardiac arrest event.

| □ Fully agree | □ Partially agree | □ Neutral | □ Partially disagree | □ Fully disagree |
| --- | --- | --- | --- | --- |

1. I have specific suicidal plans since the cardiac arrest event.

| □ Fully agree | □ Partially agree | □ Neutral | □ Partially disagree | □ Fully disagree |
| --- | --- | --- | --- | --- |

**Questionnaire on heart failure symptoms / activity of daily and professional life**

1. Do you feel episodes of dyspnea during your daily life?

| □ Yes | □ No |
| --- | --- |

1. Do you feel dyspnea after exertion?

| □ Yes | □ No |
| --- | --- |

If yes:

| □ At rest | □ During minimal exertion (e.g. get up from bed) | □ During moderate exertion (e.g. carrying home shopping bags; using stairs for one floor) | □ During strong exertion (using stairs for two to three floors) |
| --- | --- | --- | --- |

1. I regularly feel tired and exhausted rapidly.

| □ Fully agree | □ Partially agree | □ Neutral | □ Partially disagree | □ Fully disagree |
| --- | --- | --- | --- | --- |

1. I regularly feel dizzy.

| □ Fully agree | □ Partially agree | □ Neutral | □ Partially disagree | □ Fully disagree |
| --- | --- | --- | --- | --- |

1. Do you regularly recognize sweating, dizziness and/or tachycardia?

| □ Fully agree | □ Partially agree | □ Neutral | □ Partially disagree | □ Fully disagree |
| --- | --- | --- | --- | --- |

1. a. Did you faint since the cardiac arrest event?

| □ Yes | □ No |
| --- | --- |

b. If „Yes“: How often did you faint since the cardiac arrest event? _____ times.

1. a. Do you recognize tachycardia or palpitations?

| □ Yes | □ No |
| --- | --- |

b. If „Yes“: How often do you recognize tachycardia or palpitations ?

____ times per day/ week/ month

1. Do you regularly recognize chest pain after exertion / at rest?

| □ Yes | □ No |
| --- | --- |

1. I have swollen feet and-/or ankles.

| □ Fully agree | □ Partially agree | □ Neutral | □ Partially disagree | □ Fully disagree |
| --- | --- | --- | --- | --- |

1. Do you go to the toilet for more than two times at night?

| □ No | □ One time | □ Two times | □ Three times | □ > Three times |
| --- | --- | --- | --- | --- |

1. If I sleep with a flat upper body, I regularly feel shortness of breath.

| □ Fully agree | □ Partially agree | □ Neutral | □ Partially disagree | □ Fully disagree |
| --- | --- | --- | --- | --- |

1. My physical ability is as good as prior to the cardiac arrest event.

| □ Fully agree | □ Partially agree | □ Neutral | □ Partially disagree | □ Fully disagree |
| --- | --- | --- | --- | --- |

1. My physical ability regularly limits my activities of daily living.

| □ Fully agree | □ Partially agree | □ Neutral | □ Partially disagree | □ Fully disagree |
| --- | --- | --- | --- | --- |

1. My physical ability regularly limits my professional life.

| □ Fully agree | □ Partially agree | □ Neutral | □ Partially disagree | □ Fully disagree |
| --- | --- | --- | --- | --- |

| ***Supplemental Table 1.*** Characteristics of patients at follow-up examination in the post-ECPR outpatient care centre | | | | | | |
| --- | --- | --- | --- | --- | --- | --- |
| **Treatment** | | | **VA-ECMO (N=15)** | **ECMELLA (N=18)** | **Total**  **(N=33)** | **P-Value** |
| **Baseline vital signs** | | | | | | |
| **Blood pressure (mmHg)** | | | 121 (20.7)/  75.5 (12.7) | 104 (24.9)/  67.1 (22.0) | 112 (24.0)/  71 (18.3) | 0.066 / 0.216 |
| **Heart rate (beats per minute)** | | | 75.6 (13.1) | 72.0 (11.8) | 73.7 (12.4) | 0.429 |
| **Peripheral oxygen saturation (percent)** | | | 98.0  [97.0-99.0] | 99.0  [96.5-100.0] | 98.0  [97.0-100] | 0.508 |
| **12-lead electrocardiogram** | | | | | | |
| **Sinus rhythm** | | | 13 (86.7%) | 14 (77.8%) | 27 (81.8%) | 0.947 |
| **Atrial fibrillation or flutter** | | | 1 (6.7%) | 0 (0.0%) | 1 (3.0%) | 0.334 |
| **Pacemaker rhythm** | | | 1 (6.7%) | 2 (11.1%) | 3 (9.1%) | 0.164 |
| **(In-)complete left or right bundle branch block** | | | 2 (13.3%) | 3 (16.7%) | 5 (15.2%) | 0.693 |
| **Repolarisation disturbance in any ECG lead*** | | | 0 (0.0%) | 2 (11.1%) | 2 (6.1%) | 0.164 |
| **Transthoracic echocardiography** | | | | | | |
| **LVEF (percent)** | | | 51.1 (8.89) | 50.7 (9.14) | 50.9 (8.87) | 0.908 |
| **Global longitudinal strain** | | | -15.0 (5.00) | -11.9 (4.68) | -13.2 (4.96) | 0.16 |
| **Any wall motion abnormalities** | | | 4 (26.7%) | 11 (61.1%) | 15 (45.5%) | 0.081 |
| **Any severe valvular insuffiency** | | | 6 (40.0%) | 8 (44.4%) | 14 (42.4%) | 0.84 |
| **Baseline medication** | | | | | | |
| **Any anti-platelet therapy or anticoagulation** | | | 12 (80.0%) | 15 (83.3%) | 27 (81.8%) | 0.116 |
| **Aspirin** | | | 7 (46.7%) | 13 (72.2%) | 20 (60.6%) | 0.041 |
| **P2Y receptor antagonist** | | | 5 (33.3%) | 7 (38.9%) | 12 (36.4%) | 0.467 |
| **Oral anticoagulation** | | | 5 (33.3%) | 2 (11.1%) | 7 (21.2%) | 0.266 |
| **Any heart failure medication** | | | 10 (66.7%) | 14 (77.8%) | 24 (72.7%) | 0.116 |
| **ACE inhibitor or AT1 receptor blocker** | | | 9 (60.0%) | 14 (77.8%) | 23 (69.7%) | 0.059 |
| **Beta blocker** | | | 7 (46.7%) | 12 (66.7%) | 19 (57.6%) | 0.102 |
| **Digitalis or digitoxin** | | | 1 (6.7%) | 0 (0.0%) | 1 (3.0%) | 0.367 |
| **Aldosterone antagonist** | | | 3 (20.0%) | 8 (44.4%) | 11 (33.3%) | 0.102 |
| **SGLT2 inhibitor** | | | 2 (13.3%) | 2 (11.1%) | 4 (12.1%) | 0.616 |
| **Diuretic medication** | | | 4 (26.7%) | 6 (33.3%) | 10 (30.3%) | 0.456 |
| **Calcium channel blocker** | | | 1 (6.7%) | 1 (5.6%) | 2 (6.1%) | 0.616 |
| **Lipid lowering medication** | | | 7 (46.7%) | 10 (55.6%) | 17 (51.5%) | 0.334 |
| **Anti diabetic medication (except SGLT2 inhibitors)** | | | 0 (0.0%) | 2 (11.1%) | 2 (6.1%) | 0.211 |
| **Heart failure symptoms** | | | | | | |
| **Dyspnoea during daily life** | | | 8 (53.3%) | 12 (66.7%) | 20 (60.6%) | 0.454 |
| **Orthopnoea** | | 1  2  3  4  5 | 0 (0%)  2 (13.3%)  1 (6.7%)  1 (6.7%)  11 (73.3%) | 3 (16.7%)  0 (0%)  0 (0%)  3 (16.7%)  11 (61.1%) | 3 (9.1%)  2 (6.1%)  1 (3.0%)  4 (12.1%)  22 (66.7%) | 0.168 |
| **NYHA class** | | 1  2  3  4 | 5 (33.3%)  3 (20.0%)  6 (40.0%)  1 (6.7%) | 8 (44.4%)  2 (11.1%)  6 (33.3%)  2 (11.1%) | 13 (39.4%)  5 (15.2%)  12 (36.4%)  3 (9.1%) | 0.811 |
| **Rapid exhaustion in daily life** | | 12  3  4  5 | 3 (20.0%)  5 (33.3%)  2 (13.3%)  2 (13.3%)  3 (20.0%) | 6 (33.3%)  3 (16.7%)  2 (11.1%)  2 (11.1%)  5 (27.8%) | 9 (27.3%)  8 (24.2%)  4 (12.1%)  4 (12.1%)  8 (24.2%) | 0.783 |
| **Angina pectoris at exertion or at rest** | | | 5 (33.3%) | 6 (33.3%) | 11 (33.3%) | 1.0 |
| **Dizziness** | | 1  2  3  4  5 | 2 (13.3%)  7 (46.7%)  1 (6.7%)  1 (6.7%)  4 (26.7%) | 4 (22.2%)  4 (22.2%)  1 (5.6%)  2 (11.1%)  6 (33.3%) | 6 (18.2%)  11 (33.3%)  2 (6.1%)  3 (9.1%)  21 (63.6%) | 0.705 |
| **Tachycardia or palpitations** | | | 8 (53.3%) | 3 (16.7%) | 11 (33.3%) | 0.032 |
| **Combination of sweating, dizziness and/or tachycardia** | | 1  2  3  4  5 | 1 (6.7%)  3 (20.0%)  2 (13.3%)  2 (13.3%)  7 (46.7%) | 1 (5.6%)  3 (16.7%)  1 (5.6%)  3 (16.7%)  9 (50.0%) | 2 (6.1%)  6 (18.2%)  3 (9.1%)  5 (15.2%)  16 (48.5%) | 0.91 |
| **Syncope** | | | 1 (6.7%) | 0 (0.0%) | 1 (3.0%) | 0.334 |
| **Leg oedema** | | 1  2  3  4  5 | 4 (26.7%)  2 (13.3%)  3 (20.0%)  2 (13.3%)  4 (26.7%) | 4 (22.2%)  1 (5.6%)  2 (11.1%)  0 (0%)  10 (55.6%) | 8 (24.2%)  3 (9.1%)  5 (15.2%)  2 (6.1%)  14 (42.4%) | 0.318 |
| **Nycturia** | | 1  2  3  4  5 | 6 (40.0%)  4 (26.7%)  3 (20.0%)  0 (0%)  2 (13.3%) | 3 (16.7%)  5 (27.8%)  2 (11.1%)  6 (33.3%)  1 (5.6%) | 9 (27.3%)  9 (27.3%)  5 (15.2%)  6 (18.2%)  3 (9.1%) | 0.134 |
| **EQ-5D-5L visual analogue scale** | | | 52.5  [42.8-67.5] | 55.0  [38.8-66.3] | 52.5  [40.0-67.5] | 0.763 |
| **NT-proBNP (ng/L)** | | | 1260 (2290) | 947 (1680) | 1100 (2370) | 0.725 |
| **Iron level (µmol/L)** | | | 33.5 (29.3) | 26.5 (31.1) | 30.1 (29.8) | 0.543 |
| **Transferrin saturation (percent)** | | | 23.4 (21.1) | 24.6 (10.7) | 24.0 (16.5) | 0.844 |
| **Creatinine kinase (U/L)** | | | 119 (57.0) | 111 (76.2) | 114 (67.6) | 0.761 |
| **Creatinine kinase-muscle brain type (U/L)** | | | 23.1 (10.1) | 24.7 (26.6) | 24.1 (21.3) | 0.861 |
| **pH (venous blood gas)** | | | 7.34 (0.061) | 7.31 (0.101) | 7.32 (0.085) | 0.367 |
| **Partial pressure of oxygen**  **(mmHg, venous blood gas)** | | | 43.3 (15.4) | 45.3 (42.5) | 44.4 (31.8) | 0.866 |
| **Partial pressure of carbon dioxide (mmHg, venous blood gas)** | | | 43.6 (5.65) | 48.0 (7.69) | 45.9 (7.04) | 0.084 |
| **Cardiovascular risk factors** | | | | | | |
| **Low-density lipoprotein cholesterol (mg/dL)** | | | 102 (65.5) | 65.6 (29.4) | 83.1 (52.6) | 0.065 |
| **High-density lipoprotein cholesterol (mg/dL)** | | | 62.3 (41.4) | 52.1 (22.4) | 57.0 (32.8) | 0.41 |
| **Triglyceride (mg/dL)** | | | 125 (57.5) | 131 (72.9) | 128 (64.5) | 0.826 |
| **Lipoprotein a (nmol/L)** | | | 96.8 (101) | 69.1 (85.8) | 83.5 (93.3) | 0.432 |
| **Haemoglobin A1C (percent)** | | | 39.1 (7.90) | 41.3 (11.6) | 40.2 (9.82) | 0.541 |
| **Cognitive function** | | | | | | |
| **Cerebral Performance Categories scale** | | 1  2  3 | 14 (93.3%)  1 (6.7%)  0 (0.0%) | 15 (83.3%)  0 (0.0%)  1 (5.6%) | 29 (87.9%)  1 (3.0%)  1 (3.0%) | 0.285 |
| **Modified Rankin scale** | | | 1.00  [0.0-2.0] | 1.00  [0.75-1.0] | 1.00  [0.75-2.0] | 0.832 |
| **Mini Mental State Examination** | | | 29.0  [28.0-30.0] | 28.0  [26.5-30.0] | 29  [28.0-30.0] | 0.111 |
| **Stroke or TIA since ECPR event** | | | 1 (6.7%) | 3 (16.7%) | 4 (12.1%) | 0.38 |
| **Similar concentration as prior to ECPR event** | | 12  3  4  5 | 4 (26.7%)  0 (0.0%)  3 (20.0%)  6 (40.0%)  2 (13.3%) | 7 (38.9%)  2 (11.1%)  4 (22.2%)  3 (16.7%)  2 (11.1%) | 11 (33.3%)  2 (6.1%)  7 (21.2%)  9 (27.3%)  4 (12.1%) | 0.445 |
| **Trouble to memorise new information** | | 1  2  34  5 | 2 (13.3%)  5 (33.3%)  3 (20.0%)  1 (6.7%)  4 (26.7%) | 3 (16.7%)  5 (27.8%)  3 (16.7%)  1 (5.6%)  6 (33.3%) | 5 (15.2%)  10 (30.3%)  6 (18.2%)  2 (6.2%)  10 (30.3%) | 0.988 |
| **Trouble to remember old information** | | 1  2  3  45 | 0 (0%)  5 (33.3%)  1 (6.7%)  3 (20.0%)  6 (40.0%) | 2 (11.1%)  1 (5.6%)  2 (11.1%)  3 (16.7%)  10 (55.6%) | 2 (6.1%)  6 (18.2%)  3 (9.1%)  6 (18.2%)  16 (48.5%) | 0.217 |
| **Trouble to make plans** | | 1  2  3  4  5 | 1 (6.7%)  3 (20.0%)  2 (13.3%)  4 (26.7%)  5 (33.3%) | 1 (5.6%)  5 (27.8%)  1 (5.6%)  4 (22.2%)  7 (38.9%) | 2 (6.1%)  8 (24.2%)  3 (9.1%)  8 (24.2%)  12 (36.4%) | 0.924 |
| **Trouble to organise my daily life** | | 1  2  3  4  5 | 2 (13.3%)  4 (26.7%)  2 (13.3%)  2 (13.3%)  5 (33.3%) | 4 (22.2%)  3 (16.7%)  0 (0.0%)  4 (22.2%)  7 (38.9%) | 6 (18.2%)  7 (21.2%)  2 (6.1%)  6 (18.2%)  12 (36.4%) | 0.468 |
| **Mental capabilities** | | 1  2  3  4  5 | 0 (0.0%)  1 (6.7%)  8 (53.3%)  6 (40.0%)  0 (0.0%) | 2 (11.1%)  1 (5.6%)  9 (50.0%)  1 (5.6%)  5 (27.8%) | 2 (6.1%)  7 (21.2%)  2 (6.1%)  7 (21.2%)  5 (15.2%) | 0.0336 |
| **Psychologic symptoms** | | | | | | |
| **Depressive symptoms** | | 1  2  3  4  5 | 5 (33.3%)  5 (33.3%)  1 (6.7%)  0 (0.0%)  4 (26.7%) | 5 (27.8%)  2 (11.1%)  2 (11.1%)  5 (27.8%)  4 (22.2%) | 10 (30.3%)  7 (21.2%)  3 (9.1%)  5 (15.2%)  8 (24.2%) | 0.171 |
| **Thoughts that it would be better to stop being alive** | | 1  2  3  4  5 | 1 (6.7%)  2 (13.3%)  1 (6.7%)  2 (13.3%)  9 (60.0%) | 3 (16.7%)  3 (16.7%)  1 (5.6%)  3 (16.7%)  8 (44.4%) | 4 (12.1%)  5 (15.2%)  2 (6.1%)  5 (15.2%)  17 (51.5%) | 0.879 |
| **Precise suicidal thoughts** | | 1  2  3  4  5 | 1 (6.7%)  1 (6.7%)  0 (0.0%)  1 (6.7%)  12 (80.0%) | 1 (5.6%)  1 (5.6%)  1 (5.6%)  1 (5.6%)  14 (77.8%) | 2 (6.1%)  2 (6.1%)  1 (3.0%)  2 (6.1%)  26 (78.8%) | 0.926 |
| **Blood examination** | | | | | | |
| **Creatinine (mg/dL)** | | | 1.11 (0.494) | 1.21 (0.485) | 1.16 (0.483) | 0.598 |
| **Glomerular filtration rate (ml/min)** | | | 77.3 (31.2) | 73.7 (19.5) | 75.5 (25.6) | 0.707 |
| **Alanine-aminotransferase (U/L)** | | | 39.9 (52.7) | 33.0 (24.1) | 36.4 (40.4) | 0.651 |
| **Aspartat-aminotransferase (U/L)** | | | 32.8 (31.3) | 46.4 (72.1) | 39.6 (55.1) | 0.511 |
| **Gamma-glutamyl transferase (U/L)** | | | 66.1 (135) | 215 (539) | 143 (399) | 0.299 |
| **Bilirubin (mg/dL)** | | | 0.477 (0.279) | 0.615 (0.341) | 0.546 (0.314) | 0.234 |
| **Alkaline phosphatase (U/L)** | | | 148 (219) | 129 (127) | 138 (176) | 0.776 |
| **Lipase (U/L)** | | | 36.4 (12.6) | 70.7 (133) | 52.3 (90.9) | 0.373 |
| **Amylase (U/L)** | | | 58.4 (18.7) | 69.5 (41.5) | 63.3 (30.6) | 0.428 |
| **Thyroid stimulating hormone (mU/L)** | | | 1.51 (0.663) | 1.20 (0.561) | 1.36 (0.623) | 0.18 |
| **Triiodothyronine (µg/L)** | | | 1.30 (0.545) | 1.13 (0.473) | 1.21 (0.502) | 0.436 |
| **Free tetraiodothyronine (ng/L)** | | | 13.8 (2.21) | 14.5 (3.88) | 14.1 (3.15) | 0.544 |
| **Haemoglobin (g/dL)** | | | 13.9 (1.44) | 14.2 (1.45) | 14.0 (1.43) | 0.55 |
| **Leukocytes (x10^9^/L)** | | | 8.01 (1.37) | 9.71 (3.97) | 8.92 (3.12) | 0.126 |
| **Thrombocytes (x10^3^/µl)** | | | 297 (97.4) | 222 (44.4) | 257 (82.0) | 0.016 |
| **C-reactive protein (mg/L)** | | | 17.8 (55.7) | 2.61 (4.33) | 9.63 (38.2) | 0.329 |
| **Procalcitonin (µg/L)** | | | 0.06  (0.063) | 0.053  (0.041) | 0.0563 (0.052) | 0.732 |
| **Partial thromboplastin time (seconds)** | | | 34.6 (15.8) | 44.5 (54.4) | 39.9 (40.9) | 0.512 |
| **International normalised ratio** | | | 1.32 (0.675) | 1.12 (0.185) | 1.22 (0.496) | 0.303 |
| **Sodium (mmol/L)** | | | 139 (3.60) | 140 (2.21) | 140 (2.94) | 0.673 |
| **Potassium (mmol/L)** | | | 4.47 (0.353) | 4.45 (0.356) | 4.46 (0.349) | 0.856 |
| **Magnesium (mmol/L)** | | | 0.888 (0.086) | 0.869 (0.899) | 0.878 (0.089) | 0.553 |
| **Calcium (mmol/L)** | | | 2.39 (0.061) | 2.35 (0.124) | 2.37 (0.099) | 0.198 |
| **Phosphate (mmol/K)** | | | 1.12 (0.199) | 1.07 (0.273) | 1.09 (0.238) | 0.585 |
| **Activities of daily life** | | | | | | |
| **Limitation in private life due to reduced physical performance** | | 1  2  3  4  5 | 5 (33.3%)  6 (40.0%)  1 (6.7%)  0 (0.0%)  3 (20.0%) | 5 (27.8%)  6 (33.3%)  2 (11.1%)  1 (5.6%)  4 (22.2%) | 10 (30.3%)  12 (36.4%)  3 (9.1%)  1 (3.0%)  7 (21.2%) | 0.876 |
| **Limitation in professional life due to reduced physical performances** | | 1  2  3  4  5 | 5 (33.3%)  2 (13.3%)  0 (0.0%)  2 (13.3%)  6 (40.0%) | 7 (38.9%)  1 (5.6%)  1 (5.6%)  2 (11.1%)  7 (38.9%) | 12 (36.4%)  3 (9.1%)  1 (3.0%)  0 (0%)  4 (12.1%) | 0.83 |
| **Ability to return to profession** | Jobless | | 5 (33.3%)  3 (20.0%)  7 (46.7%) | 7 (38.9%)  3 (16.7%)  7 (38.9%) | 12 (36.4%)  6 (18.2%)  14 (42.4%) | 0.784 |
|  | Employed | |  |  |  |  |
|  | Retired | |  |  |  |  |
| **Reasons for no return to profession:** | | | | | | |
| Physical abilities | | | 3 (20.0%) | 6 (33.3%) | 9 (27.3%) | 0.401 |
| Psychological | | | 1 (6.7%) | 2 (11.1%) | 3 (9.1%) | 0.664 |
| Cognitive | | | 0 (0.0%) | 2 (11.1%) | 2 (6.1%) | 0.163 |
| Logistical reasons | | | 0 (0.0%) | 1 (5.6%) | 1 (3.0%) | 0.331 |
| Plan to return | | | 0 (0.0%) | 1 (5.6%) | 1 (3.0%) | 0.331 |
| **Similar performance in profession as prior to the ECPR event** | | 1  2  3  4  5 | 2 (13.3%)  1 (6.7%)  0 (0.0%)  3 (20.0%)  4 (26.7%) | 2 (11.1%)  0 (0.0%)  1 (5.6%)  3 (16.7%)  4 (22.2%) | 4 (12.1%)  1 (3.0%)  1 (3.0%)  6 (18.2%)  8 (24.2%) | 0.511 |
| **Barthel Index** | | | 100  [97.5-100.0] | 100  [96.3-100.0] | 100  [95.0-100] | 0.831 |
| **Same physical endurance as prior to the ECPR event** | | 1  2  3  4  5 | 1 (6.7%)  3 (20.0%)  1 (6.7%)  6 (40.0%)  4 (26.7%) | 2 (11.1%)  4 (22.2%)  1 (5.6%)  4 (22.2%)  7 (38.9%) | 3 (9.1%)  7 (21.2%)  2 (6.1%)  10 (30.3%)  11 (33.3%) | 0.838 |
| **6-minute walk test – total distance (meters)** | | | 391 (141) | 397 (153) | 394 (145) | 0.921 |
| **Timed „Up and Go“ test – total duration (seconds)** | | | 9.36 (3.52) | 10.8 (4.02) | 10.2 (3.81) | 0.305 |
| **Cycle ergometry** | | | | | | |
| **Total duration (minutes)** | | | 6.1 (2.1) | 6.5 (3.1) | 6.3 (2.65) | 0.651 |
| **Maximum power level (Watt)** | | | 106 (38.6) | 112 (39.9) | 109 (38.7) | 0.724 |
| **Maximum heart rate (beats per minute)** | | | 123 (31.7) | 117 (31.6) | 120 (31.2) | 0.609 |
| **Maximum blood pressure (mmHg)** | | | 153 (41.0)/  83.5 (20.0) | 144 (43.9)/  81.3 (20.8) | 148 (42.1)/  82.2 (20.1) | 0.621 / 0.796 |
| Values are displayed as frequency (percent), mean (standard deviation) or median interquartile range. Questionnaire scale: 1: fully agree**;** 2: partially agree**;** 3: neutral**;** 4: partially disagree**;** 5: fully disagree. *Repolarisation disturbance was defined as ST-segment elevation/depression or discordant T wave inversion. ACE, angiotensin-converting enzyme; AT1, angiotensin 1; CPR cardiopulmonary resuscitation; ECG, electrocardiogram; ECMELLA, VA-ECMO plus Impella(R); ECPR, extracorporeal cardiopulmonary resuscitation; ICU, intensive care unit; LVEF, left-ventricular ejection fraction; NT-proBNP, N-terminal prohormone of brain natriuretic peptide; SGLT2, sodium-glucose linked transporter 2; TIA, transient ischemic attack; VA-ECMO, veno-arterial extracorporeal membrane oxygenation. | | | | | | |

**References**

[1] Charlson ME, Pompei P, Ales KL, MacKenzie CR. A new method of classifying prognostic comorbidity in longitudinal studies: development and validation. J Chronic Dis 1987;40:373–83.

[2] Herdman M, Gudex C, Lloyd A, Janssen M, Kind P, Parkin D, et al. Development and preliminary testing of the new five-level version of EQ-5D (EQ-5D-5L). Qual Life Res 2011;20:1727–36. https://doi.org/10.1007/S11136-011-9903-X.

[3] Jennett B, Bond M. ASSESSMENT OF OUTCOME AFTER SEVERE BRAIN DAMAGE: A Practical Scale. Lancet 1975;305:480–4. https://doi.org/10.1016/S0140-6736(75)92830-5.

[4] Rankin J. Cerebral vascular accidents in patients over the age of 60. II. Prognosis. Scott Med J 1957;2:200–15. https://doi.org/10.1177/003693305700200504.

[5] Folstein MF, Folstein SE, McHugh PR. “Mini-mental state”. A practical method for grading the cognitive state of patients for the clinician. J Psychiatr Res 1975;12:189–98. https://doi.org/10.1016/0022-3956(75)90026-6.

[6] MAHONEY FI, BARTHEL DW. FUNCTIONAL EVALUATION: THE BARTHEL INDEX. Md State Med J 1965;14:61–5.

[7] Crapo RO, Casaburi R, Coates AL, Enright PL, MacIntyre NR, McKay RT, et al. ATS statement: guidelines for the six-minute walk test. Am J Respir Crit Care Med 2002;166:111–7. https://doi.org/10.1164/AJRCCM.166.1.AT1102.

[8] D P, S R. The timed “Up & Go”: a test of basic functional mobility for frail elderly persons. J Am Geriatr Soc 1991;39:142–8. https://doi.org/10.1111/J.1532-5415.1991.TB01616.X.

[9] Pina IL, Balady GJ, Hanson P, Labovitz AJ, Madonna DW, Myers J. Guidelines for Clinical Exercise Testing Laboratories. Circulation 1995;91:912–21. https://doi.org/10.1161/01.CIR.91.3.912.
